# Structural investigations on the mitochondrial uncouplers niclosamide and FCCP

**DOI:** 10.1002/2211-5463.13817

**Published:** 2024-05-15

**Authors:** Mei Ying Ng, Zhi Jian Song, Choon Hong Tan, Marcella Bassetto, Thilo Hagen

**Affiliations:** ^1^ Department of Biochemistry, Yong Loo Lin School of Medicine National University of Singapore Singapore; ^2^ Division of Chemistry and Biological Chemistry, School of Physical and Mathematical Sciences Nanyang Technological University Singapore; ^3^ School of Pharmacy and Pharmaceutical Sciences, College of Biomedical and Life Sciences Cardiff University UK; ^4^ Present address: ^*^Department of Cancer Biology Dana‐Farber Cancer Institute Boston MA USA

**Keywords:** FCCP, mitochondrial uncouplers, niclosamide, side effects

## Abstract

There has been renewed interest in using mitochondrial uncoupler compounds such as niclosamide and carbonyl cyanide p‐(trifluoromethoxy)phenylhydrazone (FCCP) for the treatment of obesity, hepatosteatosis and diseases where oxidative stress plays a role. However, both FCCP and niclosamide have undesirable effects that are not due to mitochondrial uncoupling, such as inhibition of mitochondrial oxygen consumption by FCCP and induction of DNA damage by niclosamide. Through structure–activity analysis, we identified FCCP analogues that do not inhibit mitochondrial oxygen consumption but still provided good, although less potent, uncoupling activity. We also characterized the functional role of the niclosamide 4′‐nitro group, the phenolic hydroxy group and the anilide amino group in mediating uncoupling activity. Our structural investigations provide important information that will aid further drug development.

## Graphical abstract

Uncoupler compounds such as niclosamide and FCCP increase fuel oxidation and are a potential treatment for obesity and hepatosteatosis. However, FCCP and niclosamide exhibit undesirable effects by inhibiting mitochondrial oxygen consumption and inducing DNA damage. Through structural investigations, we characterized the role of specific functional groups in mediating uncoupling and side effects, providing important information to aid further drug development.

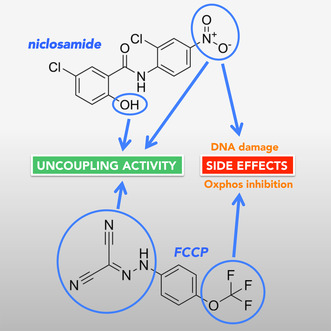

**Abbreviations**
C1‐CCP: 4‐(methoxyphenyl)carbonohydrazonoyl dicyanide
*CCCP*
: *carbonyl cyanide m‐*chlorophenylhydrazone
CCP: carbonyl cyanide phenylhydrazone
FCCP: carbonyl cyanide p‐(trifluoromethoxy)phenylhydrazone
NASH: *nonalcoholic steatohepatitis*
*ROS*
: *reactive oxygen species*
TMPD: *N*,*N*,*N*′,*N*′‐tetramethyl‐p‐phenylenediamine

There is great interest in the use of uncouplers of oxidative phosphorylation (oxphos) as potential treatment for obesity and disorders associated with oxidative stress. In different murine disease models, uncoupler compounds have shown to be highly effective treatment modalities. Moreover, the development of innovative drug delivery and prodrug activation approaches and the identification of uncouplers with a wide dynamic range have provided proof of concept that it is feasible to prevent potential side effects, thus raising the possibility of using mitochondrial uncouplers in human therapy [1, 2, 3]. Of note, a number of uncouplers are currently used in clinical trials for the treatment of obesity, diabetes, and *nonalcoholic steatohepatitis* (NASH) [4] as well as for cancer treatment [5].

Uncouplers function by dissipating the proton gradient across the mitochondrial inner membrane. Uncoupler compounds have two important chemical properties. They exist at physiological pH in a protonated and deprotonated form. Furthermore, both the protonated and the deprotonated forms of uncouplers are membrane permeable, resulting in efficient dissipation of the proton gradient. Dissipation of the proton gradient across the inner mitochondrial membrane results in increased oxygen consumption and nutrient oxidation. Under normal conditions, mitochondrial oxygen consumption and nutrient oxidation is dependent on the availability of ADP. When ATP is utilized in cells and hydrolyzed to ADP, the mitochondrial F_0_F_1_ ATP synthase utilizes the mitochondrial proton gradient to re‐phosphorylate ADP to ATP. Consumption of the proton gradient by the F_0_F_1_ ATP synthase allows for continuous proton pumping and oxidation of substrates. Uncouplers function to dissipate the proton gradient across the inner mitochondrial membrane, thereby allowing protons to enter the mitochondrial matrix without going through ATP synthase. Because in the presence of uncouplers electron transport chain activity is no longer dependent on ADP availability, oxygen consumption, and mitochondrial nutrient oxidation are maximally increased, leading to increased energy expenditure.

Mitochondrial uncouplers also lower the electron transport chain‐mediated production of reactive oxygen species (ROS), specifically of superoxide. It is believed that mitochondrial superoxide production is proportional to the half‐life of reduced intermediates of complex I and complex III in the electron transport chain [6]. A prolonged half‐life of these reduced intermediates increases the likelihood of spontaneous electron transfer to oxygen, giving rise to the superoxide radical. Superoxide is then converted to hydrogen peroxide and other more toxic ROS. By increasing the rate of electron transport, mitochondrial uncouplers decrease the half‐life of reduced complex I and complex III intermediates and lower superoxide production.

Two well‐known and highly potent uncouplers are FCCP and niclosamide. FCCP, together with its related analogue CCCP, is the most widely used uncoupler in cell‐based studies. Niclosamide was originally approved by the FDA in 1982 as an anthelmintic drug for the treatment of tapeworm infections. Recently, the drug has been considered for drug repurposing for the treatment of various diseases, including diabetes, NASH and cancer [7, 8, 9]. On the other hand, both FCCP and niclosamide are known to exert non‐specific effects that are not related to mitochondrial uncoupling. FCCP is known to inhibit oxidative phosphorylation at concentrations of 1 μm and higher [10, 11]. Niclosamide has well‐known genotoxic effects, which likely involve the aniline ring 4′‐nitro group [12]. Although numerous structural analogues of FCCP and niclosamide have been studied, the structure–activity relationship of the side effects of these uncouplers has not been explored in detail. With regards to FCCP, it is not known which functional groups contribute to the side effects and whether analogs exist with uncoupling activity but lacking side effects. With regards to niclosamide, although two prior studies have examined the role of the 4′‐nitro group, these studies were not performed using the native niclosamide structure [13] or did not measure uncoupling activity quantitatively [12].

Here we have performed a selective structural analysis to provide important novel insights. We identified FCCP analogs that do not inhibit mitochondrial oxygen consumption but still provided good, although somewhat less potent uncoupling activity. We revealed that the 4′‐nitro group in niclosamide, which has previously been shown to be critical for the genotoxic effects of the drug [12], is also important for the uncoupling activity. We also confirmed that the phenolic hydroxy group is required for the uncoupling activity of niclosamide. Finally, our results provide support for the proposed role of the anilide amino group to form a hydrogen bond with the phenolic hydroxy group, leading to increased hydrophobicity of both the neutral and the anionic form of the phenolic hydroxy group. The described studies may help in the development of novel agents with therapeutic potential while avoiding non‐specific effects.

## Materials and methods

### Synthetic chemistry methods of niclosamide and its analogues

Unless otherwise indicated, all solvents and reagents were obtained from commercial sources. All solvents used for chromatography were obtained from Fisher Scientific (Loughborough, UK) and were of HPLC grade. All reactions were performed under a nitrogen atmosphere. To record ^1^H and ^13^C‐NMR spectra, a Bruker Avance III HD spectrometer operating at 500 MHz for ^1^H and 125 MHz for ^13^C was used. Me_4_Si was utilized as internal standard. Unless otherwise indicated, deuterated chloroform was used as the solvent for NMR experiments. ^1^H chemical shifts values (δ) are referenced to the residual non‐deuterated components of the NMR solvents (δ = 7.26 ppm for CHCl_3_, etc.). The ^13^C chemical shifts (δ) are referenced to CDCl_3_ (central peak, δ = 77.0 ppm). TLC was performed on silica gel 60 F254 plastic sheets. Flash column chromatography was performed using silica cartridges in a Biotage Isolera automated system. UPLC–MS analysis was carried out on a Waters UPLC system with both Diode Array detection and Electrospray (+'ve and –'ve ion) MS detection. The stationary phase was a Waters Acquity UPLC BEH C18 1.7 μm 2.1 × 50 mm column. The mobile phase was LC–MS grade H_2_O containing 0.1% formic acid (A) and LC–MS grade MeCN containing 0.1% formic acid (B). Column temperature: 40 °C. Sample diluent: MeCN. Sample concentration 1 μg·mL^−1^. Injection volume 2 μL. Two alternative methods were used:

Linear gradient standard method (A): 90% A (0.1 min), 90–0% A (2.1 min), 0% A (0.8 min), 90% A (0.1 min); flow rate 0.5 mL·min^−1^.
Linear gradient standard method (B): 90% A (0.1 min), 90–0% A (1.5 min), 0% A (1.4 min), 90% A (0.1 min); flow rate 0.5 mL·min^−1^.

All compounds tested in biological assays were more than 95% pure. Details for the preparation and full characterization of the target final compounds are given below.

#### General method for the preparation of niclosamide (**5**) and its analogues **6**–**7**

**Fig. 1:**
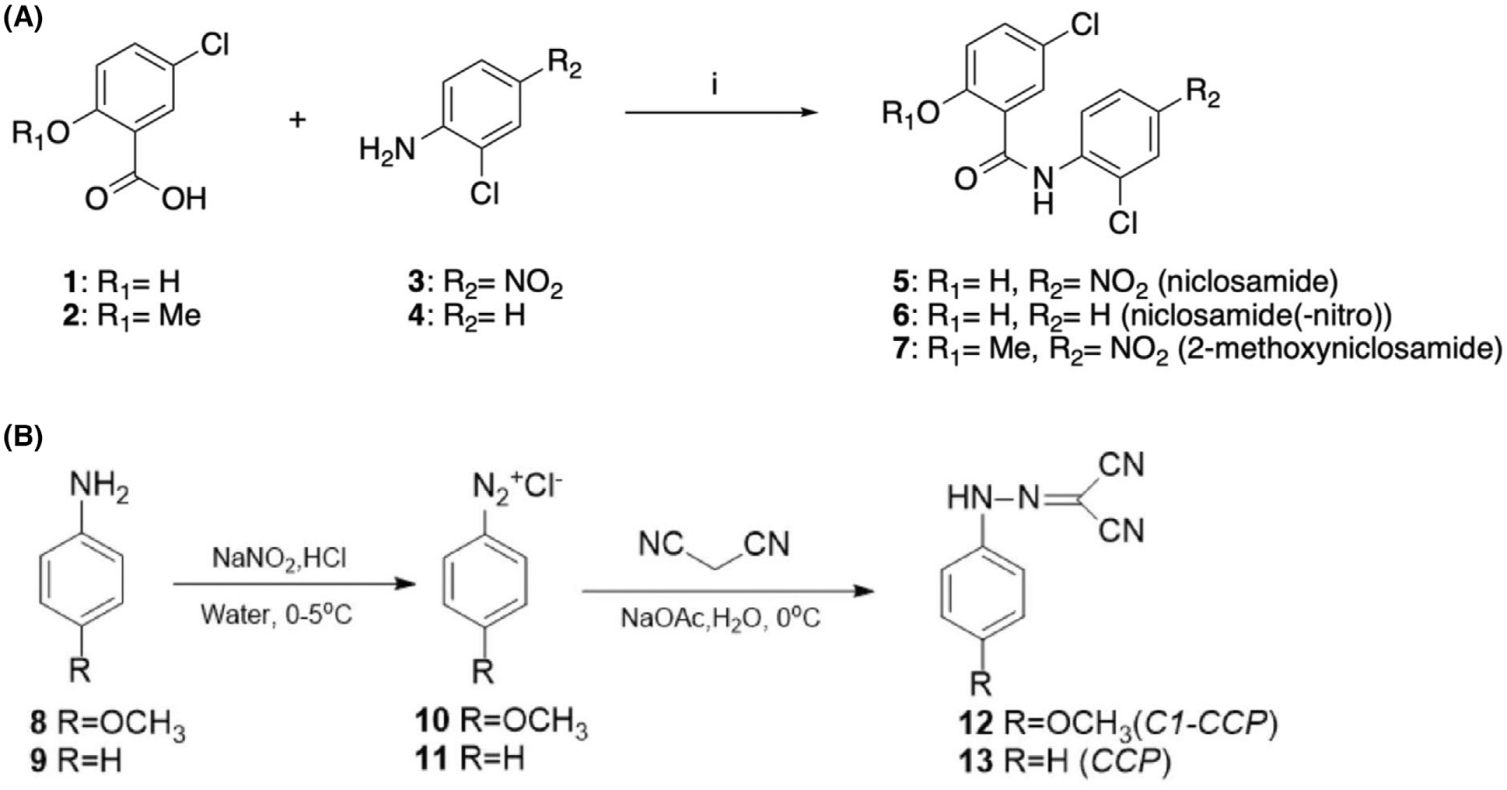
(A) *Reagents and conditions*: i. PCl_3_, PhMe, reflux, 48 h, 31–43%. Target aromatic amides **5–7** were synthesized according to a one‐step reaction. Briefly, commercially available benzoic acids **1–2** were coupled with commercial anilines **3–4**, after *in situ* conversion of the carboxylic acid functional group to the corresponding acyl chloride, using phosphorus trichloride in refluxing toluene. (B) For the synthesis of the FCCP analogs 12 and 13 see Materials and methods.

Compounds **5–7** were synthesized according to a one‐step reaction, as shown in Fig. 1. The 3‐chlorobenzoic acid derivatives **1–2** (9 mmol, 1 eq.) were dissolved in toluene (50 mL) and the appropriate 2‐chloroaniline **3–4** (9 mmol, 1 eq.) was added while stirring at room temperature, followed by phosphorus trichloride (9 mmol, 1 eq.). The mixture was stirred under reflux for 48 h. The reaction mixture was then allowed to cool to room temperature, quenched by the addition of saturated aqueous NaHCO_3_ solution, and extracted three times with ethyl acetate. The combined organic layers were washed with water and brine, dried over Na_2_SO_4_, and concentrated under *vacuum*. The crude residue was purified by automated flash column chromatography.

##### 5‐Chloro‐*N* ‐(2‐chloro‐4‐nitrophenyl)‐2‐hydroxybenzamide (**5**)

Purified by automated flash column chromatography (Biotage Isolera One, SNAP KP Sil 50 g) eluting with *n*‐hexane:EtOAc 100 : 0 v/v increasing to 0 : 100 v/v in 10 CV. Obtained in 43% yield as a pale yellow solid. ^1^H‐NMR (DMSO‐d_6_), δ: 12.33 (bs, 1H), 11.37 (bs, 1H), 8.81 (d, J = 9.2 Hz, 1H), 8.42 (d, J = 2.5 Hz, 1H), 8.29 (dd, J_1_ = 9.2 Hz, J_2_ = 2.5 Hz, 1H), 7.96 (d, J = 2.8 Hz, 1H), 7.53 (dd, J_1_ = 8.7 Hz, J_2_ = 2.8 Hz, 1H), 7.09 (d, J = 8.7 Hz, 1H). ^13^C‐NMR (DMSO‐d_6_), δ: 163.1, 155.8, 143.0, 141.7, 134.5, 130.5, 125.2, 124.3, 124.1, 122.9, 121.2, 119.9, 119.7. UPLC‐MS (Method A): R_t_ 2.35 min, MS [ESI, *m*/*z*]: 325.0, 326.9 [M‐H].

##### 5‐Chloro‐*N* ‐(2‐chlorophenyl)‐2‐hydroxybenzamide (**6**)

Purified by automated flash column chromatography (Biotage Isolera One, SNAP KP Sil 50 g) eluting with *n*‐hexane:EtOAc 100 : 0 v/v increasing to 0 : 100 v/v in 15 CV. Obtained in 35% yield as a white solid. ^1^H‐NMR (CDCl_3_), δ: 11.76 (bs, 1H), 8.46 (bs, 1H), 8.38 (dd, J_1_ = 6.7 Hz, J_2_ = 1.5 Hz, 1H), 7.54 (d, J = 2.4 Hz, 1H), 7.48 (ddd, J_1_ = 8.1 Hz, J_2_ = 1.5 Hz, J_3_ = 0.3 Hz, 1H), 7.44 (dd, J_1_ = 8.8 Hz, J_2_ = 2.4 Hz, 1H), 7.39–7.36 (m, 1H), 7.19–7.16 (m, 1H), 7.03 (dd, J_1_ = 8.8 Hz, J_2_ = 0.3 Hz, 1H). ^13^C‐NMR (CDCl_3_), δ:176.6, 160.4, 134.9, 133.3, 129.3, 127.9, 125.8, 125.0, 124.0, 123.9, 122.4, 120.5, 115.5. UPLC‐MS (Method B): R_t_ 2.00 min, MS [ESI, *m*/*z*]: 282.0. 283.9 [M + H].

##### 5‐Chloro‐*N* ‐(2‐chloro‐4‐nitrophenyl)‐2‐methoxybenzamide (**7**)

Purified by automated flash column chromatography (Biotage Isolera One, SNAP KP Sil 50 g) eluting with *n*‐hexane:DCM 50 : 50 v/v increasing to 0 : 100 v/v in 10 CV. Obtained in 31% yield as a pale yellow solid. ^1^H‐NMR (CDCl_3_), δ: 10.9 (bs, 1H), 8.97 (d, J = 9.2 Hz, 1H), 8.37 (d, J = 2.7 Hz, 1H), 8.30 (d, J = 2.5 Hz, 1H), 8.24 (dd, J_1_ = 9.2 Hz, J_2_ = 2.5 Hz, 1H), 7.54 (dd, J_1_ = 8.8 Hz, J_2_ = 2.7 Hz, 1H), 7.06 (d, J = 8.8 Hz, 1H), 4.14 (s, 3H). ^13^C‐NMR (CDCl_3_), δ: 176.6, 165.7, 146.1, 133.9, 132.4, 129.6, 127.4, 124.8, 123.7, 120.8, 116.4, 113.2, 99.9, 56.8. UPLC‐MS (Method B): R_t_ 2.23 min, MS [ESI, *m*/*z*]: 341.2, 343.1 [M + H].

### Synthetic chemistry methods of FCCP analogues C1‐CCP and CCP

The FCCP analogues (**12, 13**) were synthesized via a one‐step reaction by using the same procedure (Fig. 1B). Aniline or its derivative (**8** or **9**, 5 mmol) and concentrated HCl (4.5 mL) was added to 30 mL deionized water and then cooled to 0 °C. NaNO_2_ (350 mg, 1 eq) was dissolved in about 2 mL water and then added to the aniline mixture with stirring to obtain the intermediate compounds (**10, 11**). Malononitrile (500 mg) and sodium acetate (12.5 g) were added to 50 mL deionized water and cooled to 0 °C with stirring. The intermediate compounds were added dropwise to the malononitrile. The reaction mixture was precipitated followed by filtration, washing, and drying under vacuum.

#### 4‐(methoxyphenyl)carbonohydrazonoyl dicyanide (C1‐CCP,**12**)

C1‐CCP (**12**) was obtained in 83% yield as a yellow solid. ^1^H NMR (400 MHz, CDCl_3_) δ 10.20 (s, 1H), 7.34–7.26 (m, 2H), 7.01–6.92 (m, 2H), 3.85 (s, 3H). ^13^C NMR (100 MHz, CDCl_3_) δ 158.5, 133.6, 117.6, 115.0, 112.7, 109.0, 84.7, 55.6. MS [*m*/*z*]: 200.7, found [ESI^−^, M−H^−^]: 199.17.

#### Carbonyl cyanide phenylhydrazone (CCP,**13**)

CCP (**13**) was obtained in 81% yield as a yellow solid.^1^H NMR (400 MHz, CDCl_3_) δ 9.85 (s, 1H), 7.45 (t, J = 7.9 Hz, 2H), 7.36–7.27 (m, 3H). ^13^C NMR (100 MHz, CDCl_3_) δ 139.9, 129.9, 126.8, 116.1112.3, 108.6. MS [*m*/*z*]: 170.06, found [ESI^−^, M−H^−^] 169.02.

### Cell culture

HEK293T cells were obtained from the ATCC (CRL‐3216) and were cultured in Dulbecco's modified Eagle's medium (DMEM) supplemented with 10% fetal bovine serum, 2 mm L‐glutamine, 100 U·mL^−1^ penicillin and 100 μg·mL^−1^ streptomycin. Rho zero (ρ0) human osteosarcoma 143B cells, which lack a functional mitochondrial electron transport chain, were generated and cultured as described in King and Attardi [14]. Briefly, cells were treated with 50 ng·mL^−1^ ethidium bromide for 2 weeks and then plated at low density to isolate individual clones. ρ0 status of individual clones was confirmed by their inability to grow in the absence of uridine and the complete lack of myxothiazol‐sensitive oxygen consumption.

### Isolation of mouse liver mitochondria

Liver tissue was obtained from male C57BL/6JINV (The Jackson Laboratories, Bar Harbor, ME, USA) mice via the National University of Singapore Animal Tissue Sharing Program from a study protocol approved by the Institutional Animal Care and Use Committee (IACUC) of National University of Singapore (protocol number R21‐0892). Mouse liver mitochondria were isolated by homogenization and differential centrifugation at 4 °C in mitochondria isolation buffer [280 mm sucrose, 10 mm Tris–HCl, 1 mm EDTA (pH 7.4 at 4 °C)]. In brief, minced tissue was homogenized in mitochondria isolation buffer before centrifugation to pellet the nuclei (700 ***g***, 5 min, 4 °C). The resulting supernatant was centrifuged (9000 ***g***, 5 min, 4 °C) to pellet the mitochondria, followed by two washes and resuspending of the final mitochondrial pellet in mitochondrial isolation buffer.

### Oxygen consumption in intact HEK293T cells and isolated mouse liver mitochondria using a Clark‐type oxygen electrode

Confluent HEK293T cells were trypsinized, washed in PBS and resuspended in complete DMEM at a cell concentration of 4 × 10^6^ cells·mL^−1^. Oxygen consumption of 1 mL aliquots of the cell suspension was measured in an enclosed oxygen electrode chamber (Rank Brothers, Cambridge, UK) at 37 °C. After recording of the basal oxygen consumption rate, different additions were made through the injection port.

Oxygen consumption of mouse liver mitochondria was measured in respiration buffer [280 mm sucrose, 10 mm Tris (pH7.4), 1 mm EDTA, 2.5 mm potassium phosphate and 2.5 mm MgCl_2_] at a mitochondrial protein concentration of 0.5 mg·mL^−1^ and a temperature of 30 °C.

### Measurement of cell viability

143B ρ0 cells were plated at a density of 3000 cells per well in 96‐well plates (100 μL per well) in complete DMEM growth medium supplemented with 100 μg·mL^−1^ bromodeoxyuridine, 50 μg·mL^−1^ uridine and 1 mm pyruvate. Cells were grown for 24 h and then treated (*n* = 3) with serial dilutions of niclosamide or niclosamide(−nitro) or media (control) for 48 h. After the treatment, the cells were incubated with 0.5 mg·mL^−1^ MTT reagent (50 μL per well) for 3 h at 37 °C. Cell viability was assessed by extracting the intracellular MTT formazan in 100% dimethyl sulfoxide (DMSO) (100 μL per well) for 30 min at room temperature and measuring the absorbance at a wavelength of 540 nm. For basal cell viability controls, cells were treated (*n* = 3) with media (control) (100 μL per well) and incubated with MTT reagent 24 h after cell attachment and assayed as described above. The presented cell viability percentage was normalized to the untreated control after subtraction of basal control absorbance values. The IC50 values were calculated using graphpad prism (Insight Partners, New York City, NY, USA) equation: *Y* = 100/(1 + 10^(X‐LogIC50)^).

### Statistical analysis

Statistical analyses were carried out using an unpaired student's *t*‐test. EC50 values of uncoupling activities for the various compounds were calculated based on the dose response data using nonlinear regression in graphpad prism software.

## Results

**Fig. 2:**
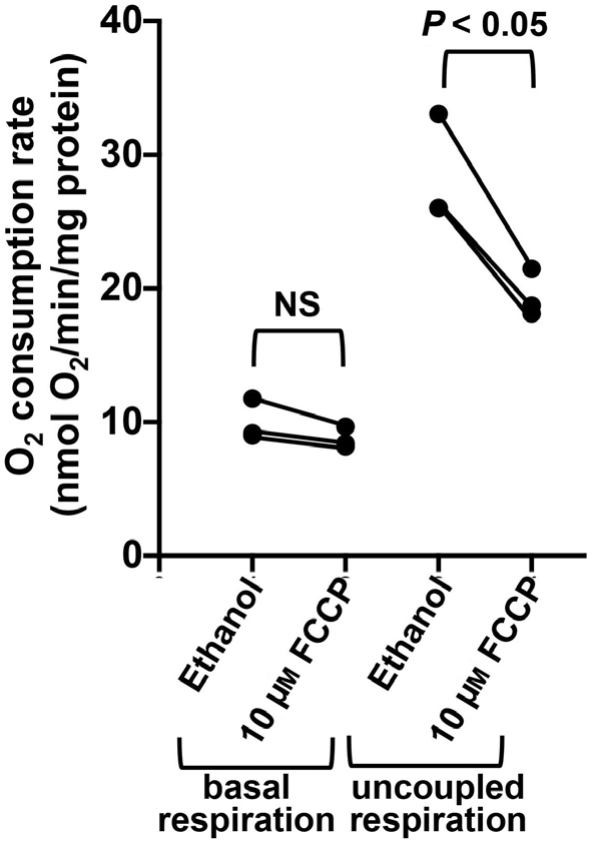
Incubation of HEK293T cells with FCCP causes sustained inhibition of oxygen consumption. HEK293T were incubated in the presence of 10 μm FCCP or ethanol (solvent used for FCCP) for 1 h. Subsequently, the FCCP containing media was removed, the cells were rinsed with PBS and trypsinized. The trypsinized cells were subjected to two washes in 20 mL PBS, followed by resuspension in 2 mL fresh DMEM. Basal oxygen consumption rates and maximum respiratory rates (upon addition of 2 μm niclosamide) were determined in duplicates using a Clark electrode. Subsequently, the protein concentration of the used cells (after removal of the complete media) was determined and oxygen consumption rates in nmol O_2_/min/mg protein were calculated. The experiment represents the average of three independent experiments. Statistical significance was determined using an unpaired *t*‐test (NS, not statistically significant).

FCCP is highly potent in dissipating the mitochondrial proton gradient, resulting in maximal stimulation of mitochondrial respiration. However, it has been reported that at high concentrations or prolonged exposure, FCCP exerts an inhibitory effect on mitochondrial oxygen consumption [10, 11]. To test whether FCCP has an inhibitory effect on mitochondrial respiration in cells and whether this effect is effect is reversible, we incubated HEK293T cells for 1 h with 10 μm FCCP. We then washed out the FCCP and trypsinized the cells, followed by measuring of the basal respiratory activity and maximum respiratory capacity using a Clark electrode. The maximum respiratory capacity was measured in the presence of the uncoupler niclosamide. Preincubation with FCCP had no effect on the basal oxygen consumption rate, but inhibited the niclosamide‐induced maximal respiratory rate by approximately 30% (Fig. 2). The results suggest that FCCP causes an irreversible inhibition of mitochondrial oxygen consumption. Preincubation with FCCP had no effect on the basal oxygen consumption rate, which is likely because under basal conditions respiration is mainly under the control of ATP demand and ADP availability.

**Fig. 3:**
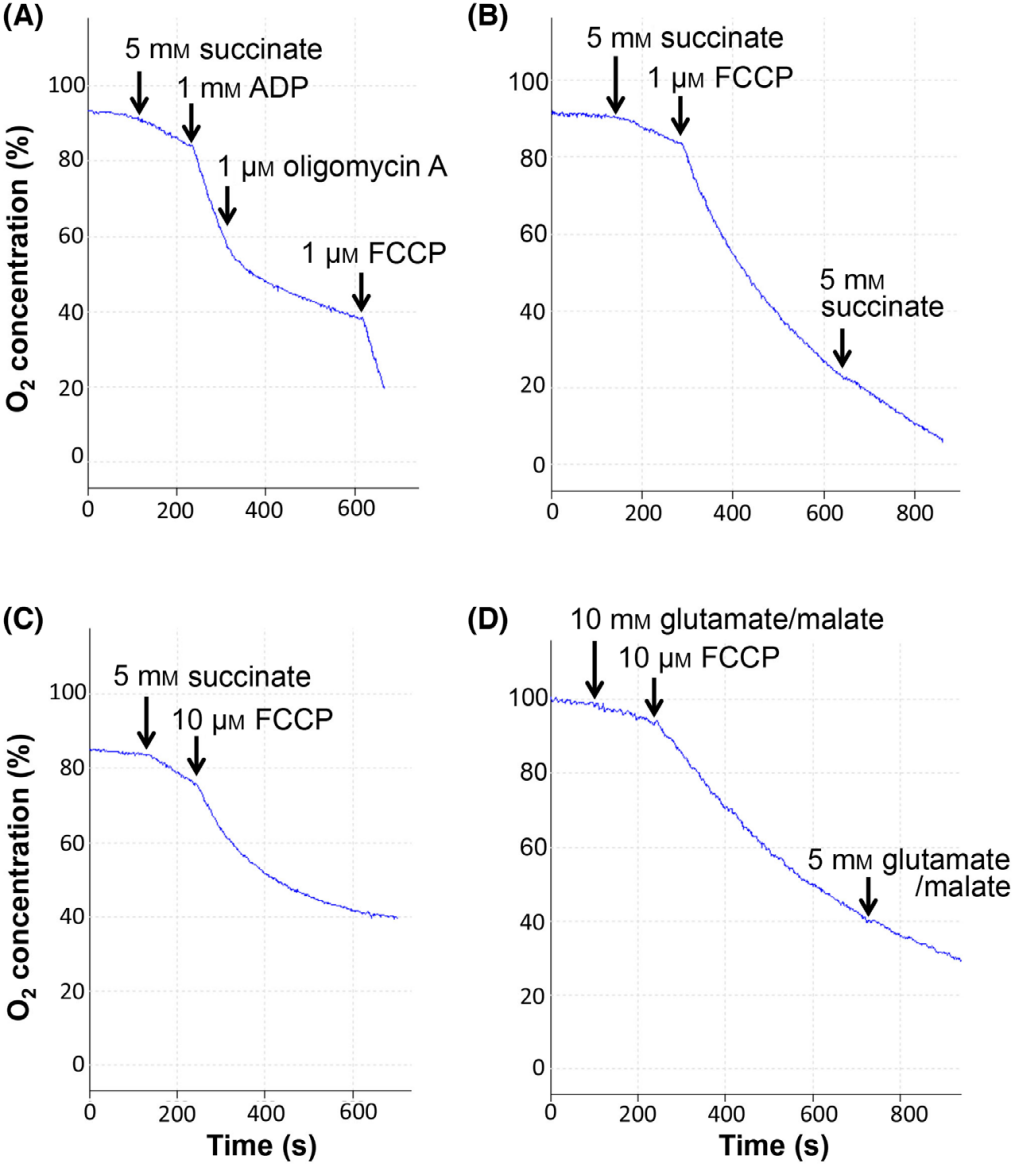
FCCP inhibits mitochondrial oxygen consumption in isolated liver mitochondria. (A) The oxygen consumption of isolated mouse liver mitochondria was measured in respiration buffer with the indicated additions through the needle access port of the Clark electrode. Stimulation of mitochondrial respiration upon addition of ADP and inhibition of respiration by 1 μm oligomycin A indicated that the mitochondria were coupled with a Respiratory Control Ratio (RCR) of 7.3. (B–D) Oxygen consumption measurement of intact isolated mouse liver mitochondria with the indicated additions. 2 μm oligomycin A was added at the beginning of the measurements. Addition of FCCP led to a gradual inhibition of mitochondrial respiration. Addition of succinate (B) or glutamate/malate (D) after FCCP did not reactivate the oxygen consumption rate, indicating that the inhibitory effect was not due to limiting amounts of substrate.

We then determined if FCCP inhibits oxygen consumption in isolated mouse liver mitochondria (Fig. 3). The isolated mitochondria fractions were functional and well coupled (Fig. 3A). Consistent with previous reports, when using succinate as a respiratory substrate, incubation of mitochondria with 1 μm FCCP led to a gradual inhibition of mitochondrial oxygen consumption over time (Fig. 3B). Incubation with 10 μm FCCP led to a more pronounced inhibitory effect (Fig. 3C). When using glutamate and malate as respiratory substrates, FCCP also exerted an inhibitory, but less pronounced effect (Fig. 3D). We hence specifically focused on the effect of FCCP on succinate‐dependent respiration.

**Fig. 4:**
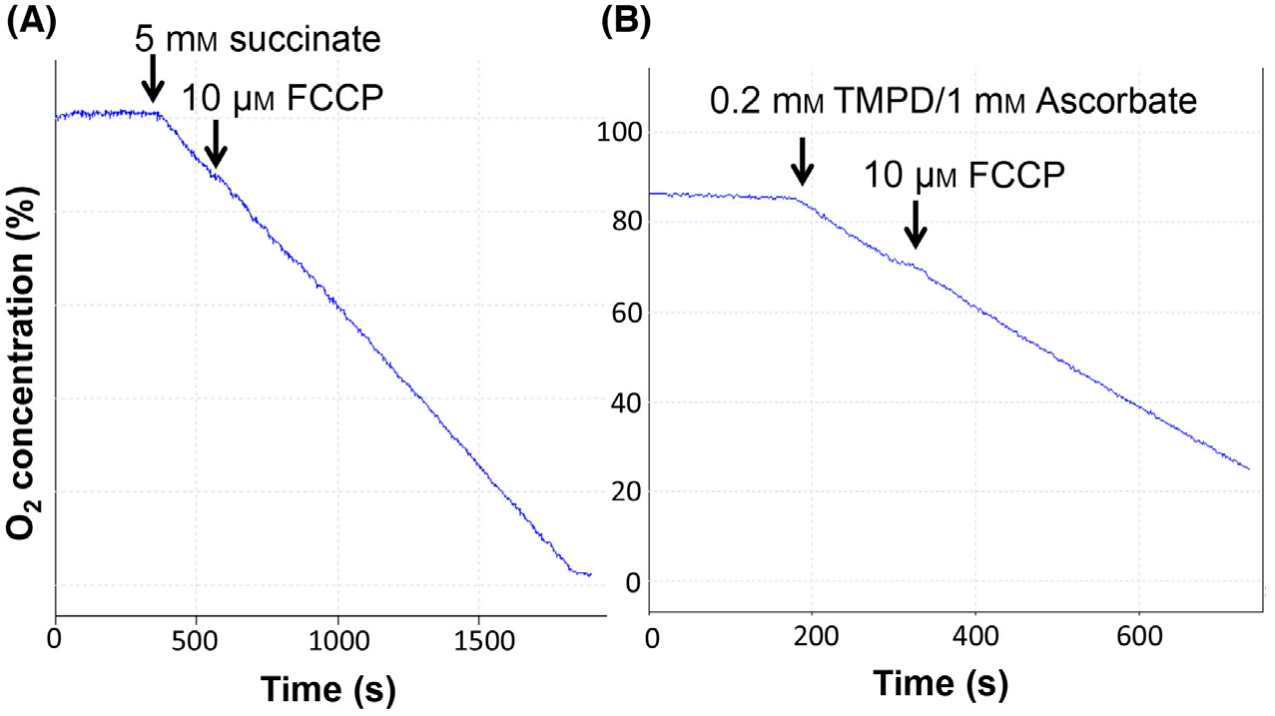
FCCP has no direct inhibitory effect on the mitochondrial electron transport chain. (A, B) Isolated mouse liver mitochondria were subjected to a freeze–thaw cycle at −20 °C and then incubated in the Clarke electrode chamber in respiration buffer. The addition of 5 mm succinate and 10 μm FCCP is indicated. (B) Intact isolated mouse liver mitochondria were incubated in respiration buffer, followed by the indicated additions of 0.2 mm TMPD (*N*,*N*,*N*′,*N*′‐tetramethyl‐p‐phenylenediamine) plus 1 mm ascorbate and 10 μm FCCP.

It has been suggested that FCCP may exert its inhibitory effect on oxygen consumption by interfering with succinate transport into the mitochondrial matrix [15, 16]. To confirm that the inhibitory effect of FCCP is dependent on mitochondrial substrate uptake, we used mitochondria that have undergone a freeze–thaw cycle and hence lack an intact inner mitochondrial membrane. The lack of an intact inner mitochondrial membrane was confirmed by the stimulation of respiration by NADH, which normally is unable to permeate the inner membrane (data not shown). In the non‐intact mitochondria, addition of 10 μm FCCP did not inhibit succinate‐dependent respiration (Fig. 4A). These results indicate that FCCP does not exert a direct inhibitory effect on the electron transport chain downstream of complex II and are consistent with an inhibitory effect of FCCP on the import of substrates into the mitochondrial matrix. We also did not observe an inhibitory effect of FCCP in intact mitochondria when we measured mitochondrial oxygen consumption in the presence of TMPD, which donates electrons directly to cytochrome c from the outside of the inner membrane (Fig. 4B), ruling out any inhibitory effect of FCCP on complex IV.

**Fig. 5:**
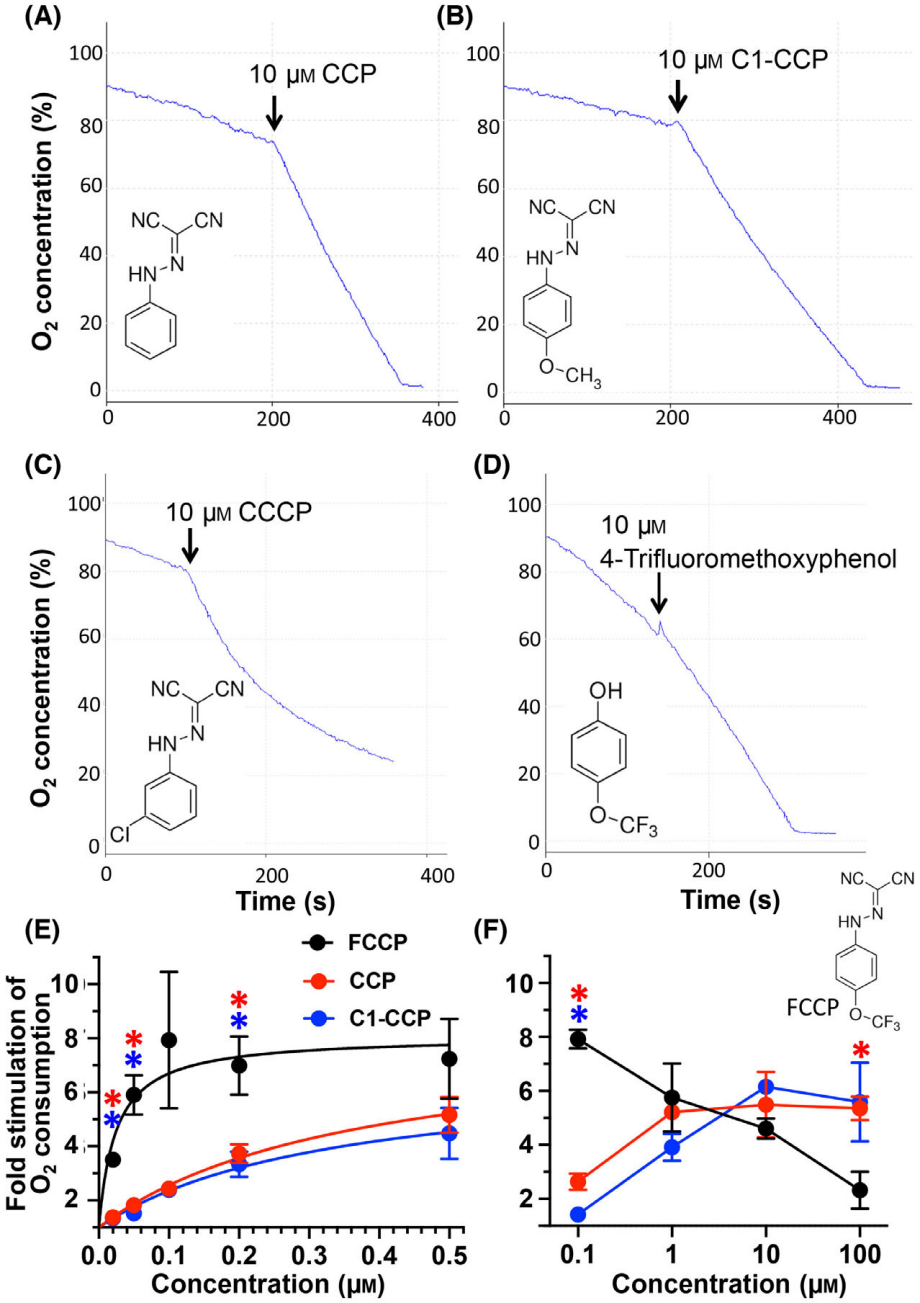
Effect of FCCP analogs on the oxygen consumption in isolated liver mitochondria. (A–D) The oxygen consumption of isolated mouse liver mitochondria was measured after addition of 5 mm succinate and 2 μm oligomycin A at time = 0. The addition of 10 μm CCP, C1‐CCP, CCCP and 4‐Trifluoromethoxyphenol is indicated. (E, F) Dose response curve of the fold stimulation of oxygen consumption of isolated mouse liver mitochondria after addition of increasing uncoupler concentrations at a low dose range (E) or a high dose range (F) compared to the basal rate (in the presence of 2 μm oligomycin A). To calculate the fold stimulation of the oxygen consumption, we used the oxygen consumption rate during the first 30s after uncoupler addition. The shown results represent the mean (±SEM) of three independent experiments. A statistical significant difference (*P* < 0.05) between the fold stimulation with CCP versus FCCP and between the fold stimulation with C1‐CCP versus FCCP at the different uncoupler concentrations is indicated with red or blue asterisks, respectively.

We were interested to explore the structure–function relationship of the inhibitory effect of FCCP on oxphos and determine if analogues with uncoupling activity but lacking the oxphos inhibitory effect can be identified. Since the uncoupling activity is mediated via the hydrazone group, we focused on the trifluoromethoxy group and synthesized an analogue that lacks this group (CCP) and one compound in which the trifluoromethoxy group was substituted with a methoxy group (C1‐CCP). Both compounds exerted only very minor inhibitory effects on mitochondrial oxygen consumption (Fig. 5A,B). This suggests that the inhibitory effect of FCCP is either directly due to the trifluoromethoxy group or is dependent on the strong electron‐withdrawing properties of the trifluoromethoxy group. The former appears unlikely, given that CCCP, which carries a strongly electron‐withdrawing chlorine group in the meta‐position, also exerted marked inhibition of mitochondrial respiration (Fig. 5C). Moreover, 4‐trifluoromethoxyphenol and 4‐trifluoromethoxyaniline, which contain the trifluoromethoxy group but lack the hydrazone group, exerted no inhibitory effect on mitochondrial oxygen consumption (Fig. 5D and data not shown), ruling out any direct effect of the trifluoromethoxy group.

Given that CCP and C1‐CCP exhibit negligible inhibitory effects on succinate‐dependent mitochondrial respiration, we determined the potency of these compounds in uncoupling oxidative phosphorylation in intact mitochondria. As shown in Fig. 5E, both CCP and C1‐CCP show reduced activity, with EC50 values of 0.38 and 0.39 μm, respectively, compared to FCCP (EC50 of 0.04 μm). We also measured the uncoupling activity of FCCP, CCP and C1‐CCP at a high dose range from 0.1 to 100 μm. The dose response curve in Fig. 5F shows that FCCP has very low uncoupling activity at a concentration of 100 μm. In contrast, CCP and C1‐CCP exhibit no major decrease in activity at very high uncoupler concentrations. In conclusion, CCP and C1‐CCP exhibit lower uncoupling activity compared to FCCP but are still very potent compared to most other commonly used uncouplers. Moreover, both uncouplers display no or only slight inhibitory effects on mitochondrial oxygen consumption even at very high concentrations.

**Fig. 6:**
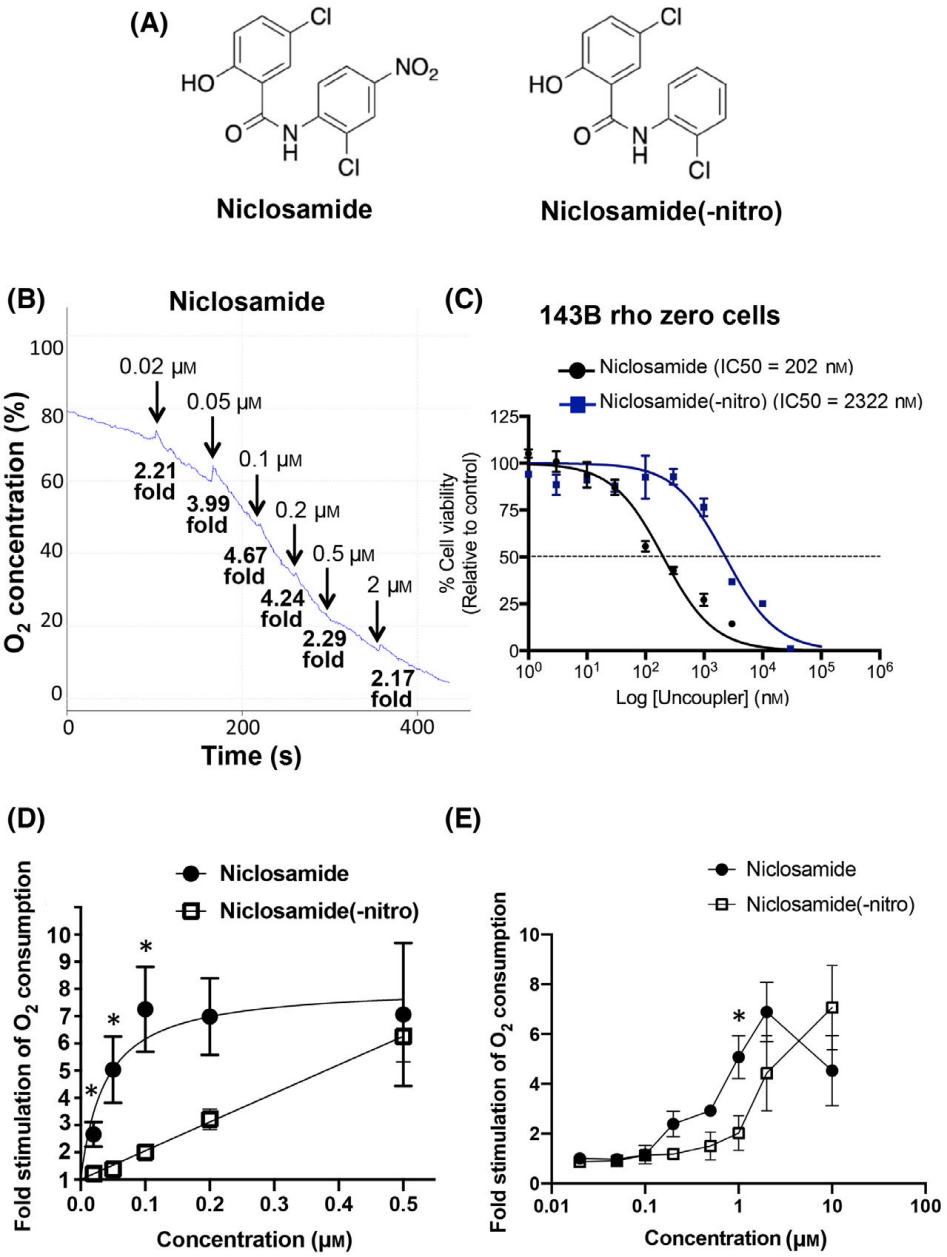
Niclosamide(−nitro) exhibits lower uncoupling activity. (A) Niclosamide(−nitro) was synthesized as described under Materials and methods. (B) Isolated mouse liver mitochondria were incubated in respiration buffer with 5 mm succinate and 2 μm oligomycin A (added at time = 0). Increasing concentrations of niclosamide were added, as indicated. The concentrations correspond to the added total concentrations. The fold stimulation compared to the basal rate is indicated. (C) Cellular toxicity in rho zero 143B cells (which lack a functional electron transport chain) was determined as described under Materials and methods. The results suggest that niclosamide toxicity is partially mediated via the aniline nitro group. (D) Isolated mouse liver mitochondria were incubated in the presence of 5 mm succinate and 2 μm oligomycin A in the Clark electrode chamber followed by the addition of different concentration of niclosamide or niclosamide(−nitro). The fold stimulation of the oxygen consumption rate after niclosamide or niclosamide(−nitro) addition compared to the basal rate was calculated and plotted. The EC50 for the uncoupling activity was calculated using nonlinear fit in graphpad prism and gave a value of 0.04 μm for niclosamide and was deemed ambiguous by the nonlinear fit analysis for niclosamide(−nitro). The graph represents the mean and SEM of three independent experiments. Statistical significance was calculated using the unpaired *t*‐test (*P < 0.05). (E) HEK293T cells were incubated in the presence of 2 μm oligomycin A in the Clark electrode chamber followed by the addition of different concentration of niclosamide or niclosamide(−nitro). The fold stimulation of the oxygen consumption rate after niclosamide or niclosamide(−nitro) addition compared to the basal rate was calculated and plotted. The calculated EC50 for the uncoupling activity was calculated using nonlinear fit in graphpad prism and gave values of 0.29 μm for niclosamide and 2.17 μm for niclosamide (−nitro). The graph represents the mean and SEM of three independent experiments (*P < 0.05).

We noted that similarly to FCCP, niclosamide when used at high concentrations also displayed an inhibitory effect on oxygen consumption in isolated mitochondria (Fig. 6A,B). In addition, niclosamide has been reported to exert cytotoxic effects, which may limit its use in chronic drug therapy. The toxic effects of niclosamide are at least partially due to the induction of DNA mutations mediated via the nitro group [17]. To study the importance of the nitro group for the activity and toxic effects of niclosamide, a niclosamide analogue lacking the nitro group was synthesized (Fig. 6A).

We initially wanted to confirm the role of the nitro group in mediating niclosamide toxicity. To rule out that differences in the cellular toxicity of niclosamide and niclosamide(−nitro) are due to their different uncoupling activities, we used ρ0 143B osteosarcoma cells, which lack a functional electron transport chain. Consistent with the recent report by Ngai *et al*. [12], we found that niclosamide(−nitro) exhibited lower toxicity compared to niclosamide (Fig. 6C).

We then assessed the uncoupling activity and cytotoxicity of this analogue. To compare the uncoupling activity of niclosamide and niclosamide(−nitro), we performed oxygen consumption measurements in both isolated liver mitochondria and intact cells. While niclosamide uncoupled oxidative phosphorylation in isolated mitochondria with an EC50 of 0.04 μm, niclosamide(−nitro) showed a markedly reduced potency compared to niclosamide with statistically significant differences between niclosamide and niclosamide(−nitro) up to 0.1 μm (Fig. 6D). Similar results were obtained in intact HEK293T cells, where the EC50 for the uncoupling activity of niclosamide(−nitro) was 2.17 μm, 7 times higher compared to niclosamide (0.29 μm) (Fig. 6E). We also noted that unlike niclosamide, niclosamide(−nitro) at high concentrations did not exhibit an inhibitory effect on oxygen consumption (Fig. 6E). Our results suggest that the nitro group plays an important role in regulating the uncoupling activity of niclosamide, likely by modifying the electron‐withdrawing properties of the aniline group on the phenolic ring (see Discussion).

**Fig. 7:**
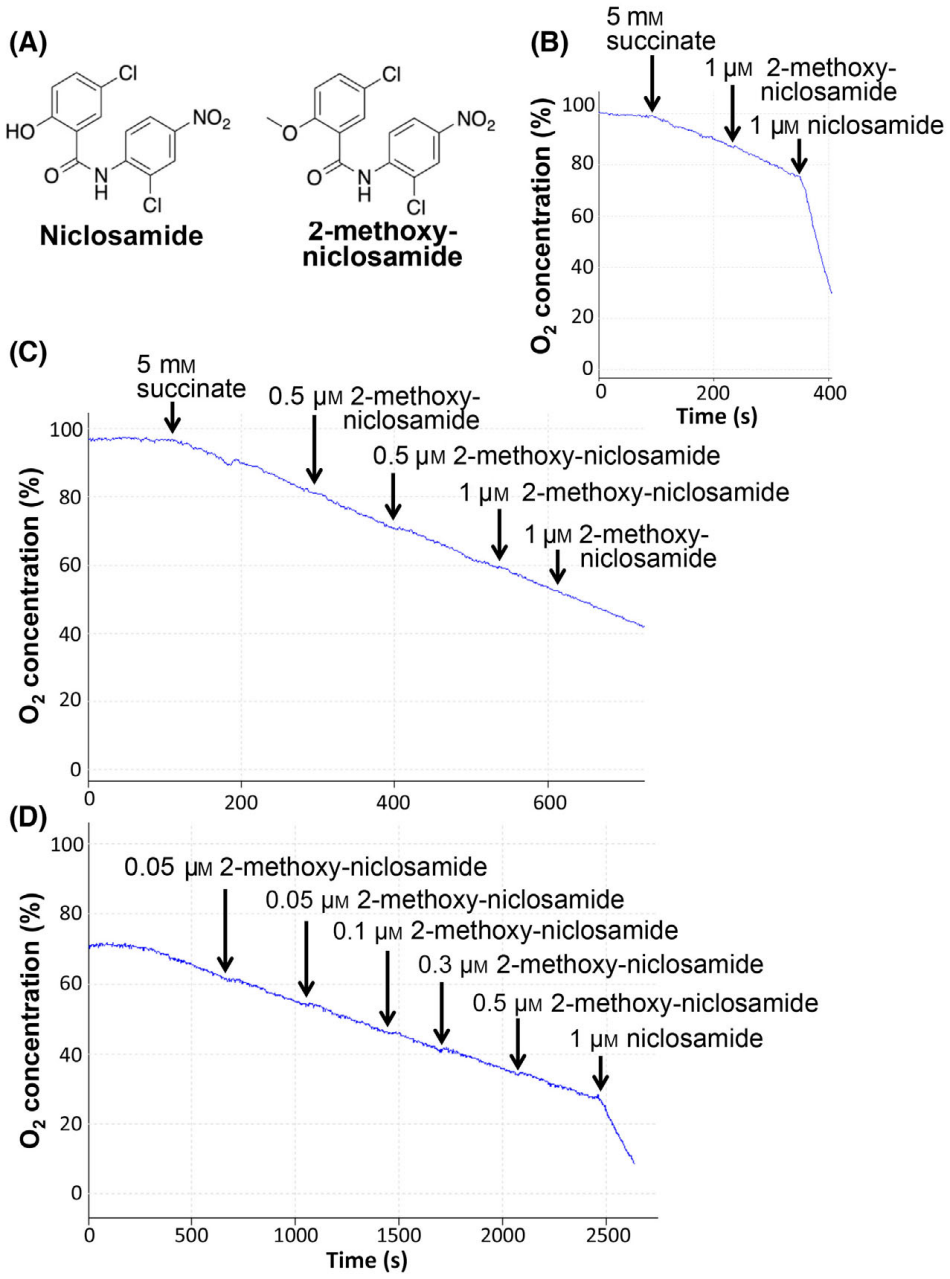
The phenolic 2‐hydroxyl group is required for the uncoupling activity of niclosamide. (A) Structure of 2‐methoxy‐niclosamide. (B, C) Intact isolated mouse liver mitochondria were incubated in the Clark electrode chamber in respiration buffer in the presence of 2 μm oligomycin A, followed by addition of the respiratory substrate succinate and increasing concentrations of 2‐methoxy‐niclosamide, as indicated. (C) HEK293T cells were incubated in complete DMEM in the Clark electrode chamber in the presence of 2 μm oligomycin A, followed by addition of 2‐methoxy‐niclosamide at increasing concentrations. The indicated concentrations in (B–D) correspond to the actual added amounts of compound. No significant uncoupling activity of 2‐methoxy‐niclosamide was observed even at concentrations up to 10 μm (results not shown).

We next wanted to determine which functional group undergoes reversible protonation and deprotonation and is hence responsible for the uncoupling activity of niclosamide. The most likely candidate is the phenolic hydroxy group. To test this, a methoxy analog was synthesized (Fig. 7A). As shown in Fig. 7B‐D, 2‐methoxy‐niclosamide completely lost uncoupling activity when using isolated mitochondria or intact cells, even when used at concentrations up to 10 μm.

**Fig. 8:**
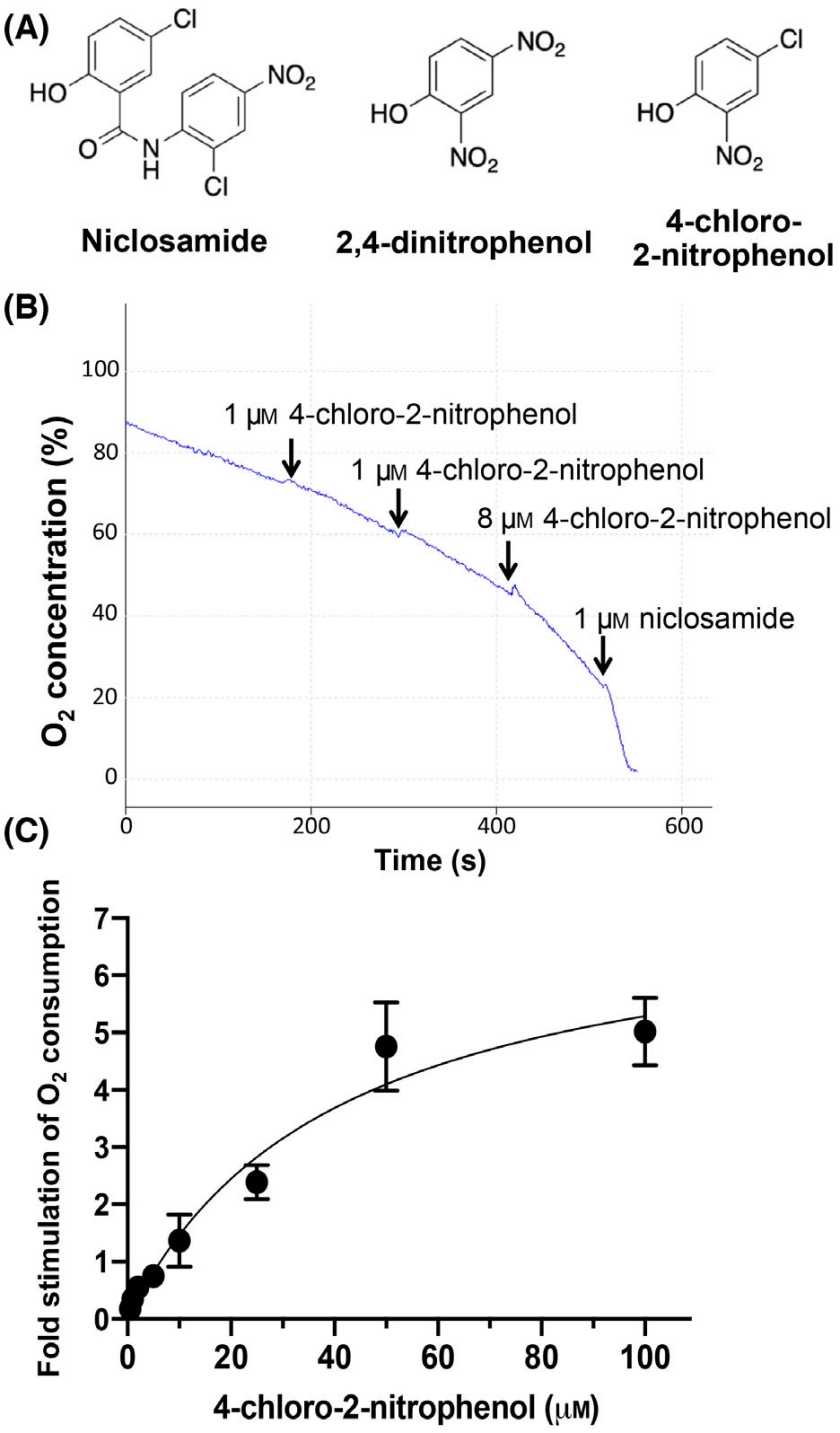
Uncoupling activity of 4‐chloro‐2‐nitrophenol. (A) Structure of 4‐chloro‐2‐nitrophenol in comparison to niclosamide and 2,4‐dinitrophenol. (B) HEK293T cells were incubated in complete DMEM in the Clark electrode chamber in the presence of 2 μm oligomycin A, followed by of increasing concentrations of 4‐chloro‐2‐nitrophenol, as indicated. The indicated concentrations correspond to the actual added amounts of compound. (C) Dose response of the uncoupling effect of 4‐chloro‐2‐nitrophenol in isolated mouse liver mitochondria. The fold stimulation of the oxygen consumption rate after 4‐chloro‐2‐nitrophenol addition compared to the basal rate (in the presence of 2 μm oligomycin A) was calculated and plotted. The data represent the averages and SEM values of three independent experiments.

With the identification of the phenolic hydroxy group as likely responsible for the uncoupling activity of niclosamide, we wanted to gain more insight into the role of the 2‐anilide group. One hypothesis is that the 2‐anilide group promotes the deprotonation of the phenolic hydroxy group by exerting an electron‐withdrawing effect, similar to the 2‐nitro group in the well‐characterized uncoupler 2,4‐dinitrophenol (Fig. 8A). Not mutually exclusive, Terada [18] proposed that a hydrogen bond between the phenolic hydroxy group and the amino group in the anilide moiety increases the hydrophobicity of both the neutral and the anionic form of the hydroxy group and promotes the delocalization of the negative charge of the anionic form of the hydroxy group. Such an effect would lead to greater permeability of both the neutral and anionic deprotonated form of niclosamide through the inner mitochondrial membrane, thus promoting uncoupling activity. To test this hypothesis, we measured the uncoupling activity of 4‐chloro‐2‐nitrophenol, in which the 2‐anilide group in niclosamide is substituted with a nitro group (Fig. 8A). The 2‐nitro group can exert strong electron‐withdrawing effects on the phenolic ring but does not interact with the phenolic hydroxy group via hydrogen bonding. 4‐chloro‐2‐nitrophenol indeed exhibited uncoupling activity (Fig. 8B,C). However, with an EC50 of 40.9 μm in isolated mitochondria, this compound was markedly less potent compared to both 2,4‐dinitrophenol (EC50 of 9.6 μm using our assay, Ng *et al*., submitted for publication) and niclosamide (EC50 of 0.04 μm, see Fig. 6D). These results provide support for the hypothesis that hydrogen bonding between the phenolic hydroxy group and the anilide amino group plays an important role in mediating the uncoupling activity of niclosamide, although alternative explanations are possible (see Discussion).

## Discussion

FCCP is the most widely used uncoupler in cell‐based studies. However, it has been recognized as early as in the 1960s that FCCP at high concentrations exerts an inhibitory effect on mitochondrial oxygen consumption [10, 11]. This can lead to various non‐specific and adverse effects in cell‐based and *in vivo* studies. For instance, Grasmick *et al*. [19] exploited the ability of uncouplers to lower mitochondrial ROS production and studied the effect of FCCP on infarction in a stroke mouse model. The authors found that FCCP inhibited basal respiration and compromised oxidative phosphorylation and contrary to their expectation, increased the infarct volume and neurological deficit.

It has been reported by van Dam [10] that when mitochondrial respiration was partially inhibited with FCCP, cytochromes and pyridine nucleotides were in the oxidized state, suggesting that substrates were not available to the mitochondrial TCA cycle dehydrogenases. Indeed, a number of studies have provided evidence that FCCP inhibits the uptake of mitochondrial substrates, in particular of succinate [15, 16]. Consistent with inhibition of substrate uptake into mitochondria, we have found that FCCP only exerts an inhibitory effect on mitochondrial oxygen consumption in intact mitochondria and cells, but not under conditions where mitochondrial substrate transport across the inner mitochondrial membrane is not required, such as when the integrity of the inner membrane is compromised or when TMPD is used as an electron donor.

The goal of this study was to characterize the structure–activity relationship of oxphos inhibition by FCCP, which has not been previously reported. Our results suggest that the trifluoromethoxy group is required, but not sufficient to exert an inhibitory effect. Analogues that lack the trifluoromethoxy group only exhibit a very minor oxphos inhibitory effect. Compounds with a trifluoromethoxy group but lacking the hydrazonyl dicyanide group exert no inhibition on oxphos. These results suggest that the removal or substitution of the trifluoromethoxy group weakens the normally strong inductive electron‐withdrawing properties of the trifluoromethoxy group and hence likely affect the pKa and reactivity of the hydrazonyl dicyanide group. The changes in inductive electron‐withdrawing properties upon removal of the trifluoromethoxy group (CCP) or substitution with a methoxy group (C1‐CCP) also lower the uncoupling activity by approximately one order of magnitude. Nonetheless, with EC50 values of 0.38 and 0.39 μm, CCP and C1‐CCP are still potent uncouplers and may be a better choice for cell‐based and *in vivo* applications.

The mechanism through which FCCP inhibits mitochondrial oxygen consumption is currently not well‐characterized. Our results show that FCCP only exerts an inhibitory effect in intact mitochondria, but not in mitochondria that lack an intact mitochondrial membrane. This result would be consistent with an inhibitory effect of FCCP on mitochondrial substrate transport. Of note, the hydrazonyl dicyanide group in FCCP has been reported to react with cysteines [20, 21, 22]. For instance, FCCP has been shown by mass spectrometry to form covalent adducts with glutathione via its strong electrophilic centre at the tertiary carbon [21]. Notably, FCCP has been reported to cause depletion of the cellular glutathione pool [23, 24]. Both the dicarboxylate and the oxoglutarate carrier can be inhibited by sulfhydryl reagents [25, 26]. While the dicarboxylate carrier mediates the net uptake of respiratory substrates such as succinate and malate into the matrix, the oxoglutarate carrier forms part of the inner mitochondrial membrane malate–aspartate shuttle. Given that the effect of FCCP appears to be at least partially irreversible (see Fig. 2). it can be speculated that FCCP inhibits mitochondrial substrate uptake via covalent adduct formation at important cysteines in mitochondrial substrate transporters. Further studies will be necessary to determine if FCCP indeed covalently modifies cysteines in mitochondrial substrate transporters.

Apart from the inhibition of oxidative phosphorylation, another major side effect of FCCP is depolarization of the plasma membrane as well as of other cellular membranes such as the lysosomal membrane [27]. Interestingly, depolarization of other mitochondrial membranes is not an inherent property of all mitochondrial uncouplers. For instance, the uncoupler BAM15 shows a preference for protonophoric activity at the mitochondrial inner membrane [28]. SHD865, a structural derivative of BAM15, was recently reported to also exhibit a significantly smaller plasma membrane depolarization compared to FCCP [29]. Finally, another recently discovered mitochondrial uncoupler, FR58P1, a bromoalkyl ester of a hydroquinone derivative, was shown to selectively dissipate the mitochondrial proton gradient without affecting plasma membrane polarization [30].

The mechanism though which these uncouplers exert a preference for protonophore activity at the inner mitochondrial membrane compared to the plasma membrane is currently unclear. In the case of BAM15 it has been suggested that the selectivity of the compound may be due to a high pKa of its ionizable proton‐releasing group. This would favor the donating of protons in the alkaline environment of the mitochondrial matrix [28]. Alternatively, BAM15 may have a structural preference for the mitochondrial inner membrane due to its unique lipid composition [28]. Similarly, the elimination or substitution of the FCCP 4‐trifluoromethoxy group in CCP and C1‐CCP, respectively, increase the pKa of the proton‐donating hydrazine group and affect the overall uncoupler structure. Thus, it would be interesting to determine if CCP and C1‐CCP also display selectivity of protonophoric activity in the mitochondrial inner membrane.

Similarly to FCCP, niclosamide at high concentrations exerted an inhibitory effect on succinate‐dependent mitochondrial oxygen consumption. As shown in Fig. 6E, a niclosamide analog lacking the aniline nitro group did not inhibit oxphos in intact cells at concentrations up to 10 μm. However, in intact isolated mitochondria, niclosamide(−nitro) also caused marked inhibition of succinate‐dependent oxygen consumption (data not shown), suggesting that oxphos inhibition is not dependent on the aniline nitro group. Of note, 2‐methoxy‐niclosamide, which has an intact aniline nitro group, but lacks the phenolic 2‐hydroxy group, exerted no oxphos inhibitory effect, suggesting that the 2‐hydroxy group is involved in the inhibition of oxphos by niclosamide. Given that the 2‐hydroxy group can exist in the deprotonated negatively charged state, the inhibitory effect of niclosamide may involve electrostatic interactions.

The major adverse effect of niclosamide is the induction of DNA damage. Niclosamide has been shown to function as a mutagen, causing DNA damage in prokaryotes as well as in mammalian cells [17, 31]. Specifically, Cortinas de Nava *et al*. [32] have shown that niclosamide causes frameshift mutations. Niclosamide has also been reported to be capable of producing clastogenic lesions (i.e. induce disruption or breakages of chromosomes) in mouse bone marrow [33] and in human lymphocytes exposed to the drug *in vitro* or *in vivo* ^*23*^. Finally, it was reported that in *Aspergillus nidulans*, niclosamide can generate mitotic crossing‐over and nondisjunctions [34]. As such, niclosamide has been proposed as a novel approach for the treatment of cancer [35, 36]. At the same time, the drug may also function as a carcinogen, which would be a concern when considering the use of niclosamide for long‐term treatment.

In support for an important role of the aniline nitro group in mediating the niclosamide‐induced DNA damage, it has been reported that in *Salmonella typhimurium* the mutagenicity of niclosamide requires nitroreductase activity as well as further metabolism [17]. In mammals nitroreductase activity is found in the liver endoplasmic reticulum as well as in the cytosol [37, 38, 39, 40] and is likely also required for the mutagenicity of niclosamide. A critical role of the nitro group in mediating DNA damage and cytotoxicity was confirmed by Ngai *et al*. [12]. These authors found that a niclosamide analog without the nitro group exhibited significantly lower cytotoxicity compared to the parent compound.

While limiting the cytotoxicity of niclosamide is important when considering long‐term therapy, one important question is how the absence of the nitro group affects the uncoupling activity of niclosamide. Interestingly, we found that with a seven times higher EC50, niclosamide(−nitro) exhibits markedly lower uncoupling activity compared to niclosamide in intact cells. Likewise, when using isolated liver mitochondria, the uncoupling activity of niclosamide(−nitro) was severely impaired compared to the parent drug. This result is in contrast to a recent study by Ngai *et al*. [12], who reported that niclosamide and niclosamide(−nitro) have similar uncoupling activities. However, these authors used high concentrations of niclosamide (2 μm and higher) and niclosamide(−nitro) (5 μm and higher). Given our determined EC50 values of niclosamide and niclosamide(−nitro) in cells of 0.29 and 2.17 μm, respectively, the doses used by Ngai *et al*. preclude a quantitative analysis.

A number of previous reports have studied the structure–activity relationship of salicylanilides structurally related to niclosamide. Terada *et al*. [41] measured the uncoupling activity of a number of salicylanilide analogs, of which 3‐tert‐butyl‐5‐chloro‐2′‐chloro‐4′‐nitrosalicylanilide and 3,5‐dichloro‐salicylanilide are most closely related to niclosamide (systematic name: 5‐chloro‐2′chloro‐4′nitrosalicylanilide). The authors expressed the uncoupling activity as log(1/C_unc_), where C_unc_ is the minimum drug concentration required for full release of state 4 respiration. The measured uncoupling activity of 3‐tert‐butyl‐5‐chloro‐2′‐chloro‐4′‐nitrosalicylanilide was 7.50 (corresponding to a C_unc_ of 32 nM). The uncoupling activity of 3,5‐dichloro‐salicylanilide was 6.34 (corresponding to a C_unc_ of 460 nM). Although the uncoupling activity of the first analog containing the 4′‐nitro group was greater, it is important to note that both compounds are structurally different from niclosamide and differ from each other in more than only the 4′‐nitro group. The study did not compare the same analogue with and without a 4′nitro group. Hence, no direct conclusions about the role of the 4′‐nitro group can be drawn from this study.

Apart from the report by Ngai *et al*. [12], to the best of our knowledge the only other previous study determining the uncoupling activities of salicylanilide analogues that only differed in the presence or absence of the 4′‐nitro group was published by Storey *et al*. [13]. These authors compared the uncoupling activity of 3,5‐dichloro‐salicylanilide and 3,5‐dichloro‐4′nitrosalicylanilide and found similar uncoupling activities of the two analogs. However, the structures of 3,5‐dichloro‐salicylanilide and 3,5‐dichloro‐4′nitrosalicylanilide are rather different from niclosamide (i.e. 5‐chloro‐2′chloro‐4′nitrosalicylanilide). Given the potential importance of niclosamide for the treatment of various diseases, it is important to study the role of the nitro group in the context of the authentic compound.

Our finding that niclosamide(−nitro) has a markedly lower uncoupling activity compared to the parent compound have some important implications. To achieve a similar uncoupling effect, a higher concentrations of niclosamide(−nitro) would have to be administered compared to niclosamide. Mechanistically, our study indicates that the 4′‐nitro group contributes significantly to the uncoupling activity of niclosamide. In particular, removal of the 4′‐nitro group may decrease the acidity of the anilide group, resulting in a decrease in the propensity for internal hydrogen bond between the amide group and the phenolic hydroxy group, thus reducing mitochondrial uncoupling (see below). Substituting the 4′‐nitro group with other functional groups in future structural investigations may provide support for this hypothesis.

With regards to the mechanistic basis of niclosamide‐mediated proton transfer across the inner mitochondrial membrane, it has been proposed by Terada [18] that uncoupling of the niclosamide‐related compound S‐13 is due to reversible deprotonation of the phenolic hydroxy group involving the formation of an intramolecular hydrophobic hydrogen bond in the salicylanilide molecule. According to Terada [18], a hydrogen bond between the phenolic hydroxy group and the amino group in the anilide moiety increases the hydrophobicity of both the neutral and the anionic form of the phenolic hydroxy group and results in the delocalization of the negative charge of the anionic form of the hydroxy group. These effects promote the permeability of both the neutral and anionic deprotonated form of niclosamide through the inner mitochondrial membrane. However, to date there has been no experimental confirmation of this proposed mechanism.

In this study, we have modified the phenolic hydroxy group of niclosamide with a methyl group and confirmed that the phenolic hydroxy group is required for uncoupling. This has not been previously shown for niclosamide or any related salicylamide. We then tried to obtain more direct evidence for the importance of hydrogen bonding between the phenolic hydroxy group and the anilide amino group. Thus, we used an analogue in which the electron‐withdrawing anilide group is substituted with a nitro group (4‐chloro‐2‐nitrophenol). In analogy to the well‐established uncoupler 2,4‐dinitrophenol, the 2‐nitro group can exert strong electron‐withdrawing inductive and resonance effects on the phenolic ring. However, the 2‐nitro group is not expected to directly promote the deprotonation of the phenolic hydroxy group via hydrogen bonding. We found that the EC50 of the uncoupling activity of 4‐chloro‐2‐nitrophenol was 40.9 μm, significantly higher compared to 2,4‐dinitrophenol (EC50 of 9.6 μm). Thus, while the 2‐nitro group can promote potent uncoupling activity in the context of 2,4‐dinitrophenol, the 2‐nitro group is unable to substitute for the 2‐anilide group in the context of niclosamide (EC50 of 0.04 μm). These results support the hypothesis that the anilide exerts more than only an electron‐withdrawing effect on the phenolic ring, but also has a direct effect on the deprotonation and hydrophobicity of the 2‐hydroxy group. Nonetheless, it is important to note that alternative explanations are possible given that chemical properties such as the logP value and the topological polar surface area are different between niclosamide and 4‐chloro‐2‐nitrophenol.

We believe that the described structural analysis provides important insights that may aid in the design of novel, more potent and less toxic uncoupler compounds. Based on our findings, it may also be possible to develop niclosamide analogs with potent uncoupling function but lacking DNA damage inducing effects as well as niclosamide anti‐cancer analogs that cause DNA damage but lack uncoupling activity.

## Conflict of interest

The authors declare no conflict of interest.

### Peer review

The peer review history for this article is available at https://www.webofscience.com/api/gateway/wos/peer‐review/10.1002/2211‐5463.13817.

## Author contributions

MB carried out the synthesis of the niclosamide analogs. MYN and ZJS carried out and CHT supervised the chemical synthesis of the FCCP analogs. MYN and TH carried out the experiments, wrote the manuscript and prepared the figures. All authors reviewed the manuscript.

## Data Availability

The data that support the findings of this study are contained within the article.
